# LoRa Architecture for V2X Communication: An Experimental Evaluation with Vehicles on the Move

**DOI:** 10.3390/s20236876

**Published:** 2020-12-01

**Authors:** Khandaker Foysal Haque, Ahmed Abdelgawad, Venkata Prasanth Yanambaka, Kumar Yelamarthi

**Affiliations:** College of Science and Engineering, Central Michigan University, Mount Pleasant, MI 48859, USA; haque1k@cmich.edu (K.F.H.); abdel1a@cmich.edu (A.A.); yanam1v@cmich.edu (V.P.Y.)

**Keywords:** vehicle to everything (V2X), vehicle to vehicle (V2V), vehicle to infrastructure (V2I), LoRa, reliable V2X communication

## Abstract

The industrial development of the last few decades has prompted an increase in the number of vehicles by multiple folds. With the increased number of vehicles on the road, safety has become one of the primary concerns. Inter vehicular communication, specially Vehicle to Everything (V2X) communication can address these pressing issues including autonomous traffic systems and autonomous driving. The reliability and effectiveness of V2X communication greatly depends on communication architecture and the associated wireless technology. Addressing this challenge, a device-to-device (D2D)-based reliable, robust, and energy-efficient V2X communication architecture is proposed with LoRa wireless technology. The proposed system takes a D2D communication approach to reduce the latency by offering direct vehicle-to-vehicle (V2V) and vehicle-to-infrastructure (V2I) communication, rather than routing the data via the LoRa WAN server. Additionally, the proposed architecture offers modularity and compact design, making it ideal for legacy systems without requiring any additional hardware. Testing and analysis suggest the proposed system can communicate reliably with roadside infrastructures and other vehicles at speeds ranging from 15–50 km per hour (kmph). The data packet consists of 12 bytes of metadata and 28 bytes of payload. At 15 kmph, a vehicle sends one data packet every 25.9 m, and at 50 kmph, it sends the same data packet every 53.34 m with reliable transitions.

## 1. Introduction

**Figure 1:**
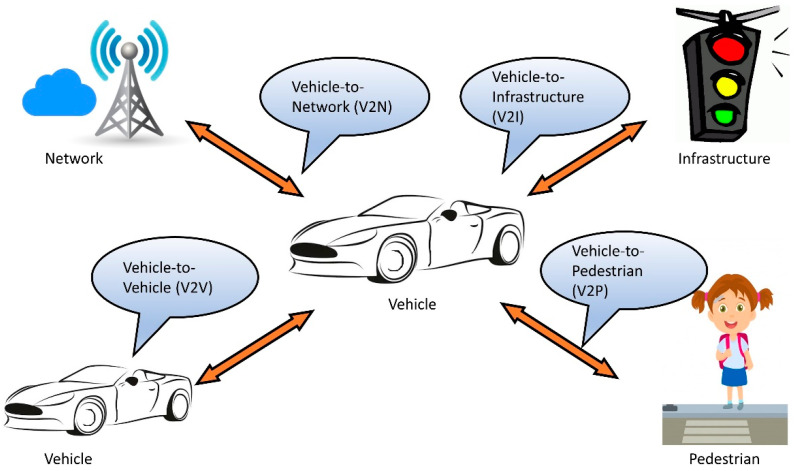
Constituents of V2X communication.

The number of autonomous vehicles on the road has been increasing rapidly over the past few years. It is imperative that the implementation of a reliable V2X communication system is created to ensure reliable and autonomous operation. Moreover, it is an integral part of the future smart cities and self-driving cars [1,2]. V2X is the communication of a vehicle to any unit, such as other vehicles or roadside infrastructures [3]. V2X communication has four main constituents including vehicle to vehicle (V2V), vehicle to infrastructures (V2I), vehicle to pedestrian (V2P), and vehicle to network (V2N) communication as presented in Figure 1. V2V is the communication among vehicles on the road, intending to avoid collisions, reduce travel time, and autonomous driving [4]. V2I communication is the exchange of data among the vehicles and road infrastructures for different applications such as collision avoidance, traffic signal control, congestion avoidance, and many more [5]. V2P is to ensure the detection of pedestrians and avoiding accidents. The underlying wireless technologies of smartphones can play a great role in setting up V2P communication. The communication among the vehicles to one or more networks, servers, or data centers is termed as V2N communication and it is one of the least explored parts of V2X communication [6].

In V2X, vehicles share their instantaneous information such as their speed, coordinates, and acceleration to other vehicles and necessary infrastructure [7]. A few of the major challenges of V2X communication include time criticality, latency intolerance, security, and privacy [8,9,10]. Building a reliable architecture and choosing the right communication technology is vital for addressing these challenges.

In V2X communication, the vehicles have an embedded system containing necessary sensors and processing units which is termed as on-board unit (OBU). OBUs are also equipped with a wireless transceiver to communicate wirelessly with other vehicles and infrastructure. On the other hand, the surrounding infrastructures are equipped with a processing unit and transceiver, combinedly termed as road side unit (RSU), capable of communicating with the vehicles. Both OBU and RSU have the capability of communicating with each other. All the vehicles carrying OBU collect the necessary data with the integrated sensors and send the data to the processing unit of the OBU. The processing unit parses, refines, and stabilize the received data and make the data packet ready to be sent by the transceiver. The vehicles and infrastructure which will receive this data are predefined by the system architecture. Upon receiving the data from other vehicles, they can warn themselves of accidents and suggest directions if necessary. Similarly, the receiving RSUs also warn and suggest a direction to the other vehicles depending on the data received by them.

One of the fundamental challenges of V2X communication is the vehicle mobility along the road and the dynamic environment. While moving along the roads, the vehicles need to perform data handover from one RSU to another RSU. The higher speed of the vehicles makes this challenging. Moreover, vehicular density and other road side infrastructures play an important role in smooth data handover, as they might affect the link quality of the communication by obstructing the direct line of sight. V2X communication aims to offer different intelligent transportation system (ITS) applications, including accident avoidance and autonomous driving, so another challenge of V2X communication is how to ensure real-time data transmission. Thus, the transmission latency should be given higher priority in a robust and reliable V2X communication architecture. Another concern regarding implementing V2X architecture is that most of the legacy vehicles do not have embedded circuitry in their ECU to facilitate V2X communication, so the modularity of the OBU and RSU is another challenge that needs to be addressed to provide V2X capability to the legacy vehicles. This is work is motivated by these challenges in reliable V2X communication. 

Extensive research has been going on with the suitability of different wireless technologies for V2X communication. Few researchers have suggested medium and long-range low-power wireless technologies like long range (LoRa) communication, Zigbee, BLE, and WIFI [11,12,13] On the other hand, researchers have adopted wireless technologies such as the Third Generation Partnership Project (3GPP) LTE and 5G new radio (NR), Wi-Max, IEEE 802.11p, and IEEE 802.16e [14,15]. 

There are different challenges associated with using these technologies also. Communication latency, transmission range, transmission throughput, and power consumption are a few important challenges that need to be considered while choosing any of those technologies for V2X [16]. WiMAX towers and 4G cell towers have a transmission range of around 50 km and 16 km, respectively [17]. However, these technologies are energy-hungry, and their frequency bands are not dedicated to V2X communication. Moreover, with the increase of the range, each gateway has to communicate with an increased number of vehicles and predict traffic and directions which would overburden the computational unit and will introduce higher latency. Zigbee is low power wireless communication technology which is based on IEEE 802.15.4 [18]. It has been vastly used in ITS applications. Zigbee gained popularity in both indoor and outdoor applications due to its low power consumption and optimized mesh networking. It allows multi-hop communication where the routing is also optimized. As it has a very low range of transmission distance (up to 100 m), so to travel the same distances with Zigbee as other wireless technology like LoRa or WiMAX can offer, it must choose multi-hop communications instead of direct D-D communications. But Zigbee can introduce higher latency and packet drops with multi-hop communication due to multiple data handover.

On the other hand, LoRa balanced between the transmission range and the data transmission rate. The transmission range of the LoRa is few kilometers with a data transmission rate of 300 bps–37.5 kbps [19]. It is also based on the low power communication standard of IEEE 802.15.4 [20]. Moreover, LoRa supports wireless area networks (WANs) through a LoRaWAN server which would be necessary for different applications of ITS. These properties of LoRa make it suitable for a simpler but efficient V2X communication which can transmit data reliably with low latency and low power consumption. One of the downsides is that it has a very low throughput, which may necessarily limit the amount of data that needs to be sent. However, 3GPP LTE and 5G NR can offer very high throughput which can even allow steaming. As this technology also offer higher throughput with lower latency, it has the potential to offer autonomous driving and an automated traffic system.

Even though substantial research is also going on in LoRa-based V2X communication, these research works have faced few challenges which include defining a robust and reliable architecture, testing the reliability and performance in real-world scenarios with actual vehicles at higher speeds. To address these existing challenges, a LoRa-based reliable and low power V2X communication architecture is proposed in this research. This work focuses on the fundamental aspects of V2X such as mobility of the vehicles, reliability of the architecture, latency, security, and energy consumption. Prototypes of the proposed architecture are designed which are based on low power microcontroller units and sensors. A performance evaluation is also conducted under practical scenarios with vehicles on the move to analyze the overall performances of the architecture. The results suggest the architecture can perform reliably under practical scenarios with vehicles on the move. It shows that LoRa presents great prospects for applications like V2X which presents mobility and a dynamic environment. The main contributions of this work are the following:

- A LoRa architecture is proposed for V2X communication to ensure higher reliability by smooth data handover from one RSU to another.
- A D2D based approach is considered in the architecture which improves the latency by enabling direct V2V and V2X communication which is missing in the literature of LoRa V2X.
- RSSI-based algorithms are proposed for V2V and V2I communication that support good link quality at a higher speed of the vehicles which is not addressed yet by the state-of-the-art literature for LoRa V2X.
- Prototypes of the OBU and RSU are designed with low power microcontrollers to maintain portability and to achieve better energy efficiency.
- The performance of the architecture is evaluated under practical scenarios with vehicles on the move at different speeds which is not addressed in the relevant literature.

The next part of the paper is organized into few sections- Section 2 of the paper describes the relevant wireless technologies for V2X and existing challenges, Section 3 describes ongoing research in the field, Section 4 depicts the proposed system architecture, Section 5 illustrates design and implementation of the system, Section 6 presents the outdoor testing and analysis and lastly, the paper is concluded in Section 7.

## 2. Wireless Technologies and the Challenges for V2X Communication

Different wireless technologies have been developed and standardize over the years for internet of things (IoT) applications like vehicular communications. These standards are regulated and maintained by organizations like IEEE, the Internet Engineering Task Force (IETF), ITU, 3GPP, Zigbee, and the LoRa Alliance [21]. These protocols are developed in different frequency bands and transmission distances. The most popular IoT wireless technologies are Zigbee, LoRaWAN, LTE-M, Z-wave, NB-IoT, BLE, IEEE 502.15.4, IEEE 802.11ah, and IEEE 802.11af [22,23].

The Zigbee standard is based on IEEE 802.15.4 for low power and low bandwidth applications. Zigbee is used in applications that involve a standalone wireless ad-hoc network with limited data transmission rate. It supports a wireless mesh network with a bandwidth ranging from 20–250 kbps depending on the transmission distances [24]. It works with the industrial, scientific, and medical (ISM) band of 2.4 GHz (global), 915 MHz (America), and 868 MHz (Europe) with transmission distances ranging from 10–100 m. This standard is widely used in low proximity monitoring and control as it is popular for its reliability, low power consumption, and cost-effectiveness. It is also used for intelligent transport systems like Embedded Middleware in Mobility Applications (EMMA) project [25]. This project has emphasized the architectural suitability of vehicle to infrastructure communication with Zigbee standard. This project has also considered and compared the performances of Bluetooth 802.15.1 and IEEE 802.11g in vehicular communications. Rhoades and Conard have considered Zigbee for Intelligent Transport Systems (ITS) in [26] for its low power consumption and rapid deployability. They have also suggested that intra-vehicular wires can be reduced by wireless transmission of the vehicle’s sensors information to the Electronic Control Unit (ECU) with Zigbee. Zigbee is also used by Fan in [27] for vehicle tracking localization. Lie et al. have also used the Zigbee standard for V2V communication and vehicle relative positioning [28]. One of the disadvantages of Zigbee is that it cannot handle a high user load as it is based on the Personal Area Network (PAN). Moreover, it has a large data overhead in comparison to LoRa which increases the latency and worsens the time criticality of the applications like V2X [29].

Unlike Zigbee, LoRa is based on wide area networks (WANs) which also operate with low power and low transmission rates. LoRaWAN is maintained by the LoRa Alliance and it operates on the sub-gigahertz ISM band of 433 MHz, 915 MHz (Australia and America), and 868 MHz (Europe) [30]. LoRaWAN offers ubiquitous connectivity both in indoor and outdoor applications with a raw maximum data rate of 27 kbps [31]. It has a transmission range of 2–5 km in urban areas whereas the range is extended up to 15 km in a suburban area with direct line-of-sight. There are three types of devices—Class A, Class B, and Class C—which have different capabilities depending on their class [32]. It is popular with long-range applications like agriculture and environmental monitoring [33,34,35], healthcare monitoring [36,37], traffic monitoring [38], localization [39,40], and various smart city applications [41]. Lora and LoRaWAN have also been vastly used in V2X communication. One of the advantages of LoRa communication is the higher transmission range, versatility, and low energy consumption. It also has some challenges like lower transmission throughput and limited bandwidth which is really a challenge for handling multiple duplex transmission.

Dedicated short-range communications (DSRC) are dedicatedly designed for vehicular communication as the name suggests. In the United States, DSRC is standardized on IEEE 802.11p which is based on IEEE 802.11 [42]. DSRC in the US also supports IEEE 1609 standards for security and internet protocol version 6 (IPv6), user datagram protocol (UDP), and transmission control protocol (TCP) for networks and transport layers. The frequency spectrum ranges from 5.8 GHz to 5.9 GHz with a transmission throughput of 2.5 Mbps. DSRC provides ITS applications through different embedded devices like RSUs, OBUs, and pedestrian devices. Extensive research is done with DSRC in [43,44] which includes a pilot project in New York City. These experiments and projects suggest their poor performances in a higher vehicular density environment. These results eventually inspired researchers to focus more on cellular vehicular communication, commonly termed as C-V2X.

LTE-based V2X communication is commonly termed as LTE-V2X and it is standardized by the 3rd Generation Partner Project (3GPP). Communication between the user equipment and the evolved node base station is supported by LTE-V2X. Rel-14 and 15 mentioned some important constituents of LTE-V2X: user equipment, V2X server, V2X control function, evolved NodeB (eNB), and multimedia broadcast multicast service [45]. Each vehicle communicates with a V2X server with unicast communication and it also broadcasts a beaconing message to all the vehicles of a single communication cell, so with LTE, V2V can exchange information with nearby UEs directly and RSU can receive this information from UEs supporting V2I applications. Moreover, this information can be transferred to any particular UE or group of UEs with V2I. The V2X server also supports V2N communication which can also communicate with any UE or group of UEs. V2V and V2P communication are also possible with infrastructure supporting V2X services like RSU [46]. Mainly two operation modes are defined in LTE-V2X in Rel 13: (i) network-based communication for the vehicles to connect with the network and (ii) direct communication mode with device-to-device communication (D2D) [47].

Though V2X developed a lot with LTE-V2X it is not enough for autonomous driving and ultra-reliable low-latency communications (URLLC). 3GPP Rel. 14 of June 2017 developed the base and paved the way for integrating 5G new Radio (NR) in V2X which is termed as NR-V2X [45]. Rel. 15 comes with phase 1 which focuses on achieving enhanced mobile broadband (eMBB) and URLLC which is the core requirement for autonomous driving whereas phase two focuses on expanding and optimizing phase 1 [47]. Moreover, NR-V2X supports the multi-cast on top of the transmission broadcast. The comparison of the discussed wireless technologies is presented in Table 1.

Moreover, there are different interesting technologies like light detection and ranging (LiDAR) which can be widely used in V2X and intelligent transportation systems (ITSs). Zhao et al. has worked on LiDAR for different V2X applications like detecting and tracking pedestrians and vehicles [53]. Lee et al. proposed a camera and LiDAR-based algorithm for predicting communication performances of multi-channel V2X [54]. Abdulla et al. have researched how LiDAR integrated with V2X can lead the way to fully autonomous driving [55]. Sualeh and Kim addressed 3D object detection and tracking with camera and LiDAR in vehicular embedded systems [56]. So, LiDAR has the potential to bring revolutionary improvements towards autonomous driving by integrating with V2X.

## 3. Related Works in LoRa V2X and Applications 

As LoRa supports long-range low power communication with decent performances in outdoor scenarios, it can play a vital role in V2X communication. Though LoRa has low transmission throughput for applications like video streaming, it is enough for necessary data transmission for applications like accident avoidance, automatic traffic signaling, congestion prediction. Due to its modularity, it can provide an excellent solution for all the legacy vehicles to be V2X enabled that are already prevalent in the roads [57], so it has inspired many researchers to focus on LoRa based vehicular communication infrastructure and applications.

Lie et al. proposed a LoRa-based V2X communication scheme and conducted performance tests with different parameter configurations [58]. This research made a point that LoRa should be configured with higher bandwidth and lower spreading factor in V2X to avoid fast fading which is caused by the Doppler effect. But this work doesn’t consider the scenarios with higher vehicular density. This research group has introduced LoRa and enhanced machine-type communication (eMTC) for V2X communication in [59]. This work simulates the bit error rate (BER) performance of LoRa under different scenarios of speed and different Doppler shifts, but this work is not tested under practical scenarios with vehicles on the move where the dynamic environment and mobility of the vehicles may hamper the communication performances. Sanchez-Iborra et al. focus on integrating LoRa in-vehicle communication using IPv6 to interconnect with future internet scenarios and evaluating its performance under real environment conditions [60]. Experiments are conducted in the V2I architectures and V2V schemes. In the V2I scenario, a suburban environment with different obstacles is evaluated. The obtained coverage is found to be much longer than the range obtained by a high-bandwidth technology, such as 4G. The V2V coverage is studied on a college campus and it is found that it covered the entirety of the campus with no shadow areas, so any incident can be reported anywhere on campus. It facilitates long-distance communication but any critical information from one vehicle to another also has to be transmitted via gateways and servers which eventually increases the latency and worsens the time criticality of the applications. One of the advantages of this architecture is that it offers reliability and a high communication range with actual vehicles on the move, but the work emphasizes only notifying traffic incidents rather than a continuous update of the vehicular information. Han et al. addressed the security issues of LoRa- based V2V and V2I communications with the physical layer key generation technique which uses RSSI values as consensus information for generating keys [61]. The proposed security system improves the key generation rate and it can reach up to 20 bit/s. Moreover, all the binary key sequences passed the statistical test suite of the National Institute of Standards and Technology (NIST). This key generation scheme is implemented with LoRa based V2V and V2I communications and tested in outdoor environments. But this model is not tested at higher speeds with actual vehicles on the road under a dynamic environment. Cheung et al. addressed the communication latency and data packet size in their research work of the V2X network with LoRa protocol [12]. This research group proposed a LoRa and LoRaWAN based system which improves the overall performances by reducing latency in V2X communications. This system has four components- end-nodes, gateways, server, and applications. Different sensors remain embedded in the end-nodes which collect data and send them to the things network (TTN) server through gateways. TTN then processes these data and route them for different applications. The system is tested and evaluated with one vehicle and four gateways. This has been tested by sending only five packets every minute, but it doesn’t show how many data packets the end-nodes can send in every unit distance, which would have presented the reliability in time-critical applications.

Many researchers have also worked on intelligent transportation systems (ITS) with LoRa. Ouya et al. designed a communication protocol with LoRa for electric vehicle charging solutions [62]. Though it offers good performances for the vehicle charging architecture, this architecture is not adequate for V2X communication. Vladimirov et al. investigated the software-defined internet of vehicles with LoRa communications in [63]. This work also proposes a laboratory test bench that facilitates simulation and field experiments under different user scenarios. The authors addressed how different nodes communicate among themselves. But the reliability of the network under different speeds and an increasing number of vehicles are ignored in this work. Santa et al. also explored vehicle monitoring platforms with low power-wide area network (LPWAN) technology LoRaWAN [64]. This work designed an end-to-end architecture for vehicle data monitoring with on-board diagnostics II (OBD-II) and a novel IETF compression scheme over LoRaWAN. The LoraWAN server also facilitates data visualization with a web-based dashboard. The system has also been tested on a university campus showing higher reliability and a packet delivery ratio of 95%. Though this work considers the mobility of the vehicles and the dynamic environment of the roads, it doesn’t mention how the higher speeds may affect the performance of the system. Janani et al. implemented V2V and V2I communication with BLE and LoRaWAN [65]. This work focuses on the development of a fundamental framework with BLE and LoRaWAN technology. It also addresses some preliminary analyses for different applications of V2X, but mobility issues are not addressed in this work. Salazar-Cabrera et al. proposed a vehicle tracking system as a proof of concept with LoRa [66]. This work shows that LoRa has the potential to address the mobility challenges for ITS applications. Moreover, this work has implemented the prototype and performed evaluations with experiments under practical circumstances. As the architecture is primarily designed for the vehicular tracking system, it is not adequate for V2X communication. A few researchers have also worked on drone-based ITS in [38] where they use drones as LoRaWAN access points and design the system for uninterrupted surveillance. But drones have higher chances of failures which may cause network holes making the system vulnerable. These state of the art LoRa V2X architectures have their respective research focus, advantages, and also the drawbacks which are depicted in Table 2. 

The table depicts the focus of the research works including their drawbacks. Most of the research works do not focus on the challenges caused by higher vehicular speeds and the dynamic environment of the roads. The key limitation in all these works is the lack of testing these systems in a real fast-paced scenario which is drastically different than indoor or outdoor testbeds under controlled environments. The higher vehicular speeds make the data handover from one RSU to another or one gateway to another more challenging. This may cause severe packet loss hampering the reliability of the communication. Moreover, most of these research works consider WAN architecture which doesn’t allow direct V2V or V2I communication, rather data is routed through the gateway to the destination. It hampers the overall latency of the communication making it unsuitable for real-time ITS applications like accident avoidance. The proposed research work aims to address these challenges by developing a D2D based reliable and low power LoRa architecture and evaluate it with vehicles on the move under practical scenarios with the dynamic environment at a higher speed of the vehicles.

## 4. Communication Algorithms of Proposed System

The performance of any low power wireless communication depends vastly on its link quality and data overheads. Link quality is measured with received signal strength and error rates. Received signal strength indicator (RSSI) of any received data depicts the quality of any wireless link. It estimates the power level of a received data from any sender or access point. Transmission throughput is another important metric that can provide the estimation of link quality for ensuring higher reliability. Some of the research works [67] have also considered throughput along with metrics like RSSI for reliable communication in vehicular networks. As throughput is beyond the scope of this work, the proposed architecture utilizes RSSI values to choose a better communication link, and for obtaining a reliable communication link with lower overheads.

As V2X presents a dynamic environment with moving OBUs along the roads, data handover from one RSU to another plays an important role. With the increased speed of the vehicles, the perfect handover becomes more challenging. Other vehicles including roadside infrastructures might also cause to prevent direct line of sight communication which makes the data handover more challenging. Thus, the proposed architecture emphasizes the smooth handover of the data from one RSU to another. LoRa communication supports WAN through the LoRaWAN server. In LoRaWAN, the data packet from any end device is sent to the server through gateways and after that, from the server, it reaches the intended destination. Routing through the server introduces higher latency which might hamper any V2X applications badly. Thus, the proposed system is based on D2D communication which reduces the communication latency by direct communication rather than unnecessary routing through the server. Moreover, the architecture is designed to facilitate modularity which can offer the legacy vehicles V2X capability without adapting any modification of the vehicles’ circuitry.

A RFM69HCW LoRa module which operates on 915 MHz and has a maximum throughput of 300 kbps with up to 100 mW of transmitting power is used as the transceiver [68]. Though it has a transmission range of 500 m in the direct line of sight, a simple quarter-wave monopole wire antenna is used in the transceiver module which might affect the range. Moreover, RSUs and OBUs will surely not be at an ideal location with a direct line of sight all the time. The research group has tested that, on a regular road with obstacles such as moving cars and pedestrians, a RFM69HCW placed at 1 m height can communicate consistently with almost no packet drop up to 160 m with an RSSI value of approximately −60 dBm. At a 40 m distance, the RSSI value improved to a value of approximately −35 dBm. 

**Figure 2:**
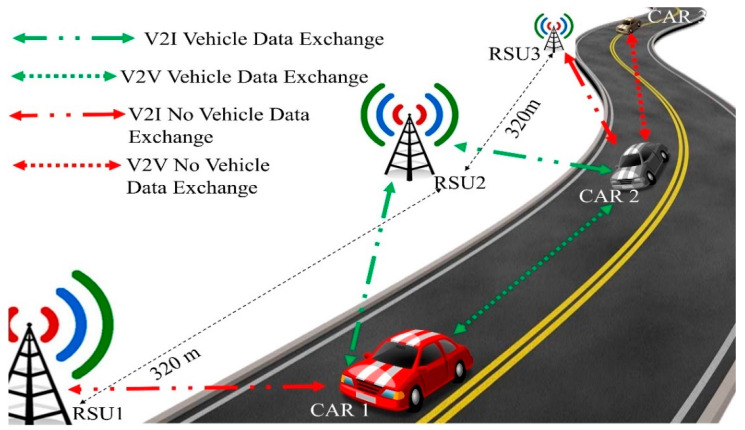
The proposed V2X communication architecture.

The proposed V2X communication architecture is presented in Figure 2. The green links denote the exchange of vehicle data whereas the red links denote that vehicles or RSUs are within the transmission range of each other but do not exchange the vehicle data according to the defined algorithms 1 & 2. While going through a road, the OBU in the car communicates with RSUs in its vicinity. CAR1 finds RSU1 and RSU2 within its transmission range whereas CAR2 finds RSU2 and RSU3 within its range. Depending on the higher RSSI value from the RSUs, CAR1 starts sending sensor data to the RSU2 and maintain sending the data to it until it reaches the threshold value of −60 dBm. At the same time, it looks for any other vehicle within its radius of 40 m. CAR1 finds CAR2 and vice-versa within the designated range and sends vehicle data to each other. Though RSU1 is within the transmission range of CAR1, it keeps sending data to the only RSU2 to reduce the data overheads. Similarly, CAR2 sends data to RSU2 only following the V2I algorithm even after having RSU3 in the transmission range. Moreover, CAR2 does not send sensor data to CAR3 as it is out of the designated perimeter. 

OBU first finds and sorts vehicles and RSUs in its vicinity. Then the sender OBU collects, parse, and sends the data to the designated vehicles and RSUs. Depending on the application, the sender OBU might also receive any necessary feedback along with the acknowledgment from the receiving units. For V2I communication, an OBU wants to reach the RSU with the best link quality instead of broadcasting the vehicle data for all the RSUs in its vicinity. This will make communication efficient and also reduce data overheads. Firstly, an OBU broadcasts a ‘Hello’ message in its transmission range. The RSUs in the vicinity receive the ‘Hello’ message and send an acknowledgment (ACK) back. Then, the OBU knows the RSSI values of all the RSUs in its vicinity. From the RSSI values, OBU knows which RSU has the better link quality to send the collected sensor data. The OBU chooses the RSU with maximum RSSI value to send the sensor data. 

The defined RSU sends an ACK upon receiving the vehicle data from the OBU. Upon receiving the ACK from the receiver RSU, OBU updates the RSSI thus checking the link quality again. The OBU keeps sending the data to that particular RSU until the RSSI value reaches a certain predefined threshold value. This threshold value depends on the distance up to which the transceiver can communicate perfectly with no packet drops under the usual traffic scenario. In this case, the LoRa transceiver that used could communicate reliably up to 160 m with no packet drops. Therefore, the RSUs are placed at a distance of 320 m from each other with a radius of 160 m each. The corresponding RSSI value at 160 m is noted as −60 dBm. Thus, the threshold RSSI is set to −60 dBm, so that OBU switches from this RSU to another as the communication becomes unreliable. When the RSSI from the receiving RSU crosses this threshold, the OBU broadcasts the ‘Hello’ message again to repeat the same procedure. By this algorithm, an OBU always remains connected to the most reliable RSU in its transmission range and it switches to another RSU only when the link quality degrades to a certain level to hamper communication link. The described V2I communication algorithm is depicted by Algorithm 1.
**Algorithm 1** Sending Data from OBU to RSU    **while** Serial reading available **do**        *VehicleData*  ←  *SensorData*        **If** radio available **then**            Broadcast to wait ‘Hello’ to RSUs            Receive ACKs from RSUs            *R_1_….R_N_*  ←  *Kalman filtered RSSI of RSU_1_….RSU_N_*            *R_max_*  ←  *maximum RSSI value from R_1_….R_N_*            *RSU_max_*  ←  *RSU with maximum RSSI*          **else**
            **print** Can’t find any RSUs          **end if**          **while**
*R_max_* > −60 **do**            Send to wait *VehicleData* to RSU_max_            Receive ACKs from RSU_max_            *R_max_*  ←  *Kalman filtered RSSI of RSU_max_*          **end while**
    **end while**


For V2V communication, an OBU broadcasts the ‘Hello’ message again for all the vehicles in transmission range as like in V2I communication. Upon receiving the data by other OBUs, they send the ACKs back and let the sender know the RSSI. When the sender OBU receives the RSSI values from other vehicles, it decides to which vehicles it will be sending the data to. OBU does not send the vehicle data to all the vehicles in the range which will make it inefficient, rather it sends the data to the closer vehicles only for making them aware of its location, direction, and acceleration. It is found that, with RFM69HCW, the RSSI value reaches −35 dBm (approximately) when the sender and the receiver are at a distance of 40 m on a regular road with usual traffic. In the proposed architecture, OBU sends the data to the vehicles within a radius of 40 m making it coherent and efficient. OBU compares the RSSI values of other vehicles to check if they are greater than −35 dBm and send the vehicle data to other vehicles within that perimeter. Algorithm 2 presents the V2V communication protocol for the proposed architecture.
**Algorithm 2** Sending Data from OBU to another OBU    **while** Serial reading available **do**        *VehicleData*  ←  *SensorData*        **If** radio available **then**            Broadcast to wait ‘Hello’ to OBUs            Receive ACKs from OBUs            *R_1_….R_N_* ← *Kalman filtered RSSI of OBU_1_….OBU_N_*            *R_1_….R_M_* ← *(R_1_….R_N_)* > − *35 (M number of OBU has RSSI greater than − 35)*            Send to wait *VehicleData* to OBU_1_….OBU_M_            Receive ACKs from OBU_1_….OBU_M_        **else**
            **print** Can’t find any OBUs        **end if**
    **end while**


It is also worth mentioning that RSSI measurement in a mobile environment is less trustworthy and fluctuates from the actual value. The Kalman filter is an optimal estimator which is also recursive to process the new measurements as they are received. It is ideal for any dynamic environment where the received values are continuously changing. Moreover, it is also quite fast which makes it suitable for real-time embedded systems. A Kalman filter is comprised of two main steps: (i) prediction step and (ii) update step. The prediction step does the prediction of the next RSSI from the earlier value whereas the update step estimates the current RSSI value from the value received at that step. Equations (1) and (2) shows the prediction step and the update step of the Kalman filter, respectively [69]. Here, X is the mean estimation of the RSSI, P is the prediction error covariance, A is the transition matrix, Q is the noise covariance matrix, B is the input effect vector and U is the control input. One of the benefits of the Kalman filter is that it only needs the memory of the previous state which makes it very light on memory, so the Kalman filter is suitable for stabilizing the RSSI values removing the outliers. Many researchers have also used the Kalman filter to stabilize the RSSI values where precise value is required for the applications:

(1) $$X_{\bar{K}}=A_{K-1}X_{K-1}+B_{K}U_{K}$$

(2) $$P_{K}=A_{K-1}P_{K-1}A_{K-1}^{T}+Q_{K-1}$$

**Figure 3:**
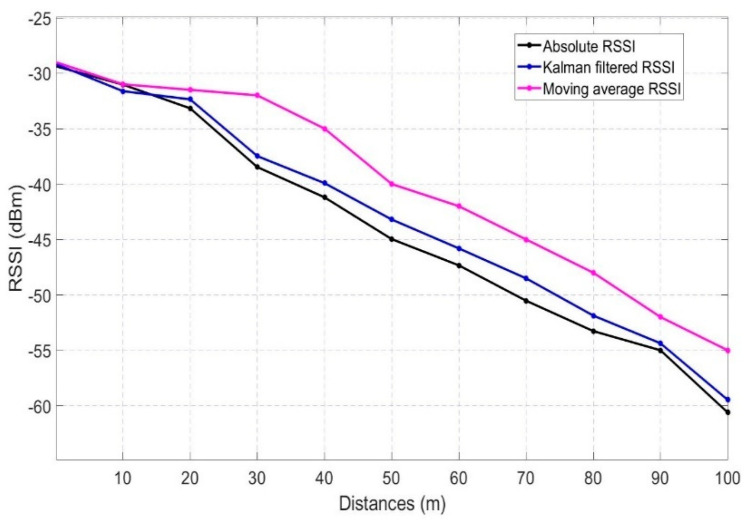
RSSI comparison of absolute, Kalman filtered values and moving average values.

The Kalman filter is computationally heavier in comparison to calculating the raw RSSI value. Few researchers have also used a moving average filter for stabilizing the RSSI values. A performance test has been carried out for finding the accuracy and stabilizing capability of the Kalman filter and moving average filter in comparison to the absolute RSSI values as in Figure 3. The average of 50 RSSI values taken at static state has been taken as the absolute RSSI value whereas the moving average filter takes 5 RSSI readings at a time for stabilizing any outlier fluctuation. The test has been carried out in a 100 m space at a speed of 5–10 kmph taking the reading in every 10 m of interval. Figure 2 shows the comparison of the moving average and Kalman filtered values with respect to the absolute values (Mean of 50 values). The results show that the Kalman filtered values get much closer to the absolute values in comparison to the moving average filter thus it stabilizes more perfectly. This is because the RSSI values change very fast for any mobile object and the moving average filter always calculates the dynamic average of any given number of points. So, with moving average filter RSSI values get update slowly and move away from the absolute value. On the contrary, the Kalman filter adapts to this dynamic environment more precisely with the prediction and update step. Thus, the Kalman filter is adopted in the proposed algorithms for better stabilization of RSSI values which is one of the factors for ensuring better reliability.

## 5. Prototype Implementation of the Proposed System 

**Figure 4:**
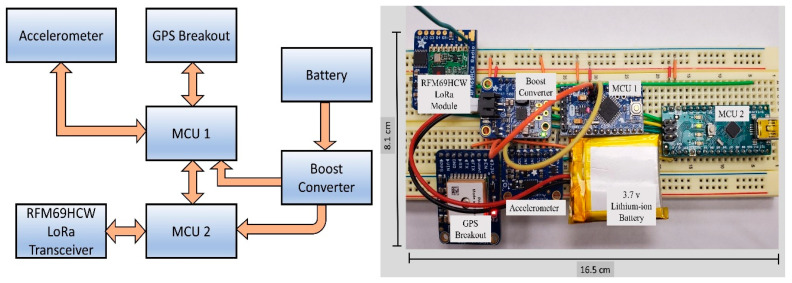
Architecture and prototype of the OBU.

**Figure 5:**
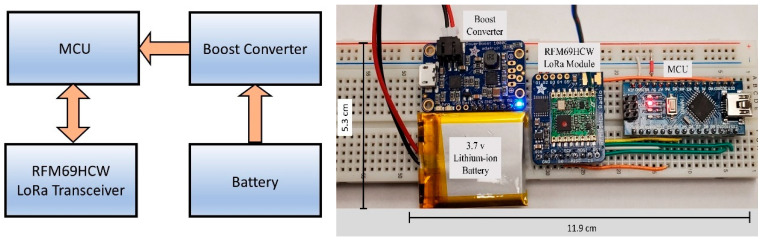
Architecture and prototype of the RSU.

Two main components of the proposed architecture are OBU and RSU. As OBUs are integrated into vehicles to collect and send vehicle data to other vehicles and infrastructures, they are equipped with sensors and transceivers. To make it computationally efficient, and maintain low power consumption, OBU is comprised of two micro-controller units (MCUs). One MCU processes and parses the sensor data and communicates serially to transfer the data to the second MCU that remains connected to the LoRa transceiver RFM69HCW module. Arduino pro mini and Arduino nano boards are used as MCU1 and MCU2 respectively and both the boards use the same ATMega328p microcontroller chip which has the static random-access memory (SRAM) of only 2 KB and a clock speed of 16 MHz ADXL345, a digital 3 axis accelerometer, and an MTK3339 chipset-based GPS module are integrated to the MCU1 with inter-integrated circuit (I2C) serial bus and software serial respectively. MCU1 gets the data from the sensors and parses them to prepare the required payload. X, Y, Z-axis acceleration data along with latitude and longitude data are sent to MCU2 with serial communication. MCU2 is integrated with the RFM69HCW transceiver module with the serial peripheral interface (SPI) bus. A TPS61090-based boost converter is used both in OBU and RSU to supply 5 V from a very compact 3.7 V Lithium-Ion battery. RSUs are much simpler in construction in comparison to OBUs. RSUs have only one MCU unit integrated with the same RFM69HCW LoRa module with the SPI bus. Figure 4 and Figure 5 show the prototypes of the OBU and RSU, respectively. MCU1 sends the collected data to MCU2 with serial communication and MCU2 being integrated with the LoRa transceiver transmits the data to other OBUs and RSUs.

**Figure 6:**
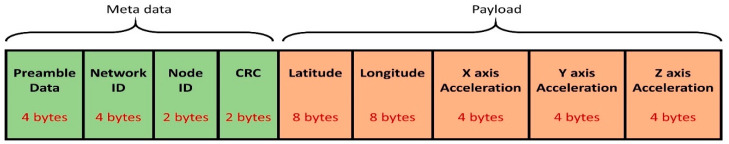
Data packet components and the respective data bytes.

**Figure 7:**
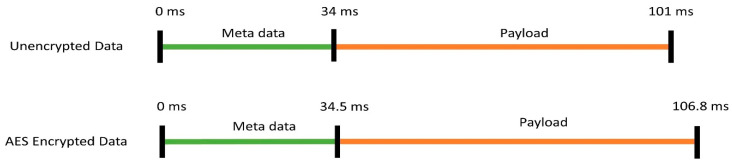
Reception time of the transmitted data packet components.

A data packet of 40 bytes is sent each time from OBU to other vehicles and infrastructures. This data packet is comprised of metadata and sensor data. Figure 6 shows all the data packet components and their respective data bytes and Figure 7 shows the time they take for the reception. The metadata, preamble data, network id, node id, and cyclic redundancy check (CRC) data takes 4, 4, 2, and 2 bytes, respectively. The reception of preamble data, network id, and node id altogether have a duration of 34 ms. The total payload is 28 bytes which are comprised of latitude, longitude, and 3-axis acceleration data. The total payload takes 67 ms to be received and processed at the receiving end for any unit.

Security is one of the major concerns for any vehicular communication. To make the communication more secure, LoRa supports advanced encryption standard (AES) 128-bit shared key encryption [70]. To address the security concerns, AES 128-bit shared key encryption is included among the deployed units. As it increases the security by encrypting the transmitted data, it also increases the reception time as the data needs to be decrypted [71]. With AES encryption, the metadata takes 34.5 ms whereas the payload takes 106.8 ms for the reception. It takes 5.8 ms for decrypting the received data by any unit with a 128-bit AES algorithm. This is the trade-off the system must consider for avoiding cyber vulnerability. 

## 6. Outdoor Testing and Analysis of the Prototypes 

### 6.1. Preliminary Handoff Testing with and without Kalman Filter

The spreading factor (SF) of LoRa plays an important role in any network deployment as the data rate, the transmission distance, time-on-air (latency) and energy consumption depend on it [72]. The RFMHCW module is used in both the OBU and RSU and they have the spreading factor from SF7-SF12. Increasing the SF would increase the transmission range significantly but that would also affect the data rate and latency. V2X has time-critical applications where higher latency can hamper the performances drastically. Moreover, higher data rates would also support the architecture to transmit more data which would be vital for applications like traffic flow optimization and optimized traffic light control [73]. Table 3 shows the SFs and the corresponding parameters of the LoRa communication for a data packet of 15 bytes over 125 kHz bandwidth. SF7 has the highest throughput of 5.47 kbps whereas with SF12 the data rate is 0.29 kbps. The time-on-air is only 36 ms (25-byte packet) with SF7 in comparison to the 682 ms by SF12, but at SF7 the signal-to-noise ratio (SNR) is limited to −6 compared to −20 at SF12. Considering these parameters, SF7 is chosen for the proposed V2X architecture to facilitate the least time-on-air and highest possible data rate with LoRa. All the field tests, experiments, and evaluations are done with SF7 at 125 kHz.

**Figure 8:**
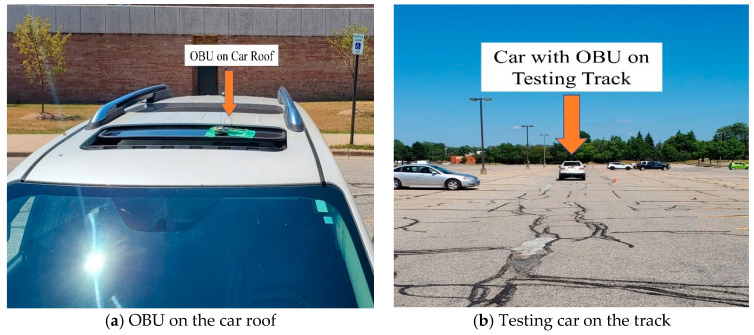
Testing setup for outdoor experiments.

Outdoor testing of the prototypes is conducted with actual cars on the road, by integrating the OBU at the car roof and connecting RSUs with computers for data analysis. Figure 8 presents the part of the testing set up with OBU on the car roof and testing the car on the track.

**Figure 9:**
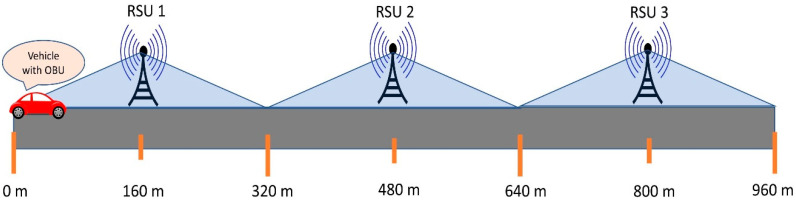
Testing setup of the track with one vehicle and three RSU.

One of the important challenges of the proposed architecture is the handoff from one RSU to another in V2I. To evaluate the handoff, the architecture is tested with multiple RSUs in a university environment. As the earlier tests suggest, RFM69HCW can communicate consistently up to 160 m in a mobile environment with regular traffic on the road. Distances between two consecutive RSUs are maintained to 320 m allowing each RSU to have a radius of 160 m. Two RSUs are placed at a distance of 320 m from each other as in Figure 9. The architecture is primarily tested with simple ‘Hello’ message, thus the data packet is consisting of only 16 bytes in comparison to generated vehicle data of 40 bytes.

Moreover, there will be no data generation time as the OBU is sending only the ‘Hello’ message to OBU which means the OBU is no needed to collect and parse the data from the sensors to send each packet data. It reduces the burden on the processing unit and sends each data packet with an interval of only (1.67 s). According to Algorithm 1, when a car goes along the road it should communicate with the RSU depending on the best link quality and should switch from that RSU to another when the link quality degrades and reaches a defined threshold value. Thus, if two RSUs are placed at an equal interval and a vehicle with OBU goes along the road, the RSUs should receive an equal number of data packets.

**Figure 10:**
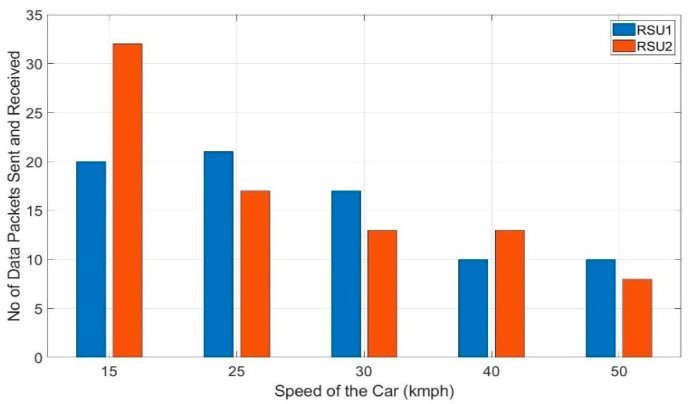
The number of data packets received by each RSU in preliminary testing without Kalman filter.

**Figure 11:**
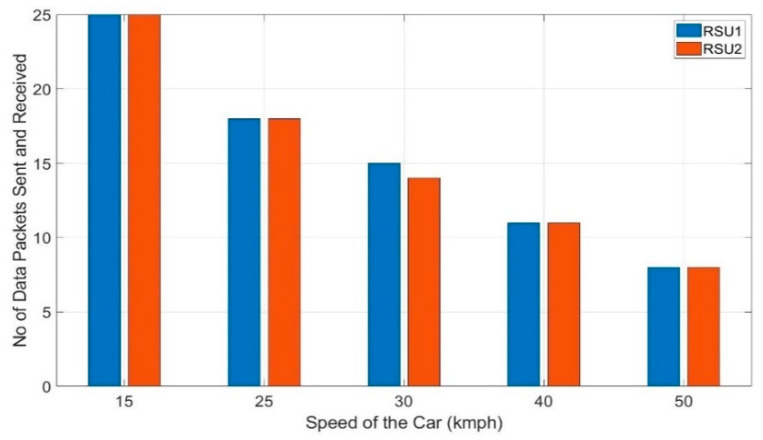
The number of data packets received by each RSU in preliminary testing with Kalman filter.

This preliminary test has been carried out first without the Kalman filter in the algorithm. The average of three trials of the test is taken into consideration in this work. Figure 10 shows the number of data packets received by each of the RSUs in this preliminary test. It shows a considerable percentage of discrepancies in the number of data packets received among the RSUs whereas according to the V2I algorithm, they should have received an equal number of data packets from the OBU. The number of data packets fluctuates by 48.75% (20 vs. 32) for 15 kmph and 21.28% (17 vs. 21) for 25 kmph. It is to note that, for 50 kmph, the fluctuation is 22.5% which is considerably lower than that of 15 kmph. So, it is evident that the fluctuation of the number of data packets received by RSU 1 and RSU 2 is not due to the higher speed of the car rather it is due to the fluctuation of the RSSI readings. As presented in Section 4, the Kalman filter is introduced in the algorithm and this preliminary test is repeated with the same setup. Figure 11 shows the number of data packets received by each RSUs with the Kalman filter in the algorithm. This shows that the handoffs between the two RSUs improved significantly due to the stabilization of the RSSI values.

At 15 kmph, fluctuation of the number of data packets improved by 48.75% and for 50 kmph, it improved by 22.5%. Even though the handoffs improved, the Kalman filter increased the computational burden. With the Kalman filter in the algorithm, it takes an interval of 2.75 s before the next packet is sent. Without the Kalman filter, it would take an interval of 2.59 s to run the algorithm once and send a data packet to the algorithm defined destination. This trade-off needs to be considered to achieve smoother handoffs and ensuring improved reliability. All the data packets that are sent from OBU are received by RSU 1 or 2 with no packet loss for both the tests- without and with Kalman filter. So, a packet delivery ratio of 100% is obtained in the preliminary tests. These results suggest that the proposed architecture would perform reliably with smooth handoffs under practical scenarios also. Further testing is needed under practical scenarios where the reliability might be affected by other vehicles, pedestrians, and buildings causing loss of direct line of sight. 

### 6.2. Testing in Real-World Scenario without and with Kalman Filter

**Figure 12:**
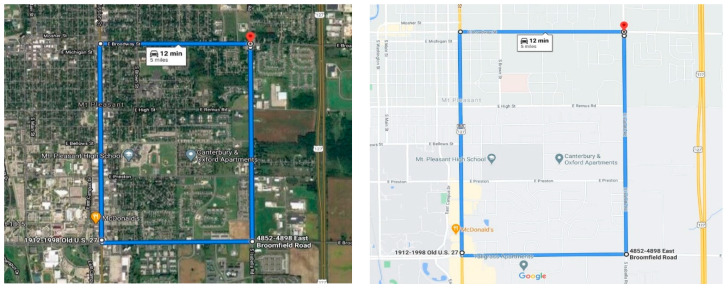
Route of the experimental evaluation.

Extensive testing is conducted with multiple RSUs placed at 320 m apart from each other with different speeds of the car to analyze the reliability of the communication. Figure 9 shows the testing setup of the track where the RSU 1, 2, and 3 are placed at 160 m, 480 m, and 800 m mark on the track at a distance of 320 m from each other. The whole setup is implemented and tested in the city of Mount Pleasant, Michigan at the campus area of Central Michigan University. Figure 12 shows the testing route of the proposed V2X architecture. The highest speed limit of the chosen road is 50 kmph, thus the architecture is evaluated with the speeds ranging from 15–50 kmph. Figure 12 also shows the starting point and the endpoint along the road. There are different infrastructures, trees, and electric poles alongside the testing route which blocked the direct line of sight from the vehicles to RSUs. 

The OBUs are placed on top of the car whereas the RSUs are placed at a height ranging from 1–1.5 m and they are connected to the computers for monitoring data packet reception. The lower height of the RSUs hampered the direct line of sight in many instances thus also affected the reliable transmission range. The higher reliable transmission range is achievable by placing the RSUs in a higher position and using UFL antennas instead of the wire antennas. The RSUs are placed at a comparatively lower height (1–1.5 m) considering RSUs need to be connected to a computer for data monitoring and there are no infrastructures along the road to place those at a higher position along the road. The car is equipped with OBU and starts from 0 m mark. According to algorithm 1, for 0–320 m, the car should send the vehicle data to RSU 1. When the car reaches the 320 m mark of the track, the car should switch from RSU1 and start sending the data to RSU2. Similarly, the car should switch from RSU2 to RSU3 when it reaches the 640 m mark. Thus, the car should get the coverage of 320 m from each of the RSUs-along 0–320 m from RSU1, along 320–640 m from RSU2, and along 640–960 m from RSU3. Thus, ideally with a smooth transition of RSUs, each RSU should receive an equal number of data points for any given speed. 

**Figure 13:**
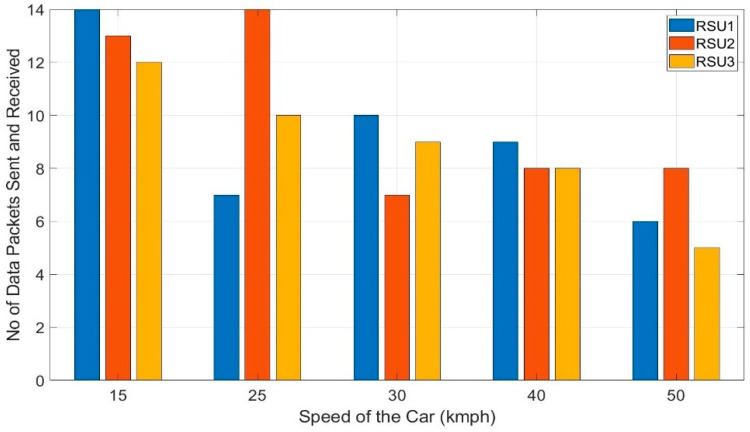
Data points received by each RSU at different speeds of the car without Kalman filter.

Most of the applications of V2X is time-critical and need instantaneous responses. It is also needed to have a continuous exchange of vehicle data with a short interval of time among vehicles and infrastructures. So, the number of data packets received by any RSU while crossing a particular distance is very important. Moreover, a smooth transition from sending data from one RSU to another RSU is also very important. These factors explain both the reliability and stability of V2X communication architecture. Tests are conducted at different speeds of the car from 15–50 kmph with multiple trials to record the number of data packets received by each RSU. At any particular speed, the test is run 3 times at different locations but with the same setup. This would randomize the factors like the visibility of the RSU, roadside infrastructure, and traffic volume on the road. The average of the three trials is taken into consideration to calculate the number of data packets received by each RSU at any particular speed. Figure 13 shows the number of data packets received by RSU1, 2, and 3 at a speed of 15, 25, 30, 40 and 50 kmph of the vehicle.

It shows the transitioning of the RSUs and the data packets received by each RSU without the Kalman filter. As RSU 1, 2, and 3 are of equal distance apart and the speed of the testing vehicle is kept constant on a single run, each of the RSUs should receive an equal number of data packets from the vehicle. But without stabilizing the RSSI values, the transition of the RSUs become more erratic.

It clearly shows that RSU 1, 2, and 3 do not receive an equal number of data packets for any of the testing speeds- 15, 25, 30, 40, and 50 kmph. This is because of the sudden fluctuation of the RSSI value as the algorithms depend on the RSSI values for switching from one RSU to another.

For 25 kmph, OBU switches from RSU1 to RSU2 very early as the fluctuated value reaches the threshold RSSI of –60 dBm and compels the OBU to switch from RSU1 to RSU2. At 25 kmph, the variation of the number of data packets received by RSUs is 53.84% which is the worst case. The number of received data packets by RSUs varies by 10.5%, 23.23%, 7.87% and 30.28%, respectively, for 15, 30, 40, and 50 kmph. This sudden switching between RSUs hampers the reliability of the architecture badly and might cause some unintended maneuvers leading to failing the whole system. This type of erratic transitioning can be avoided with the Kalman filter.

**Figure 14:**
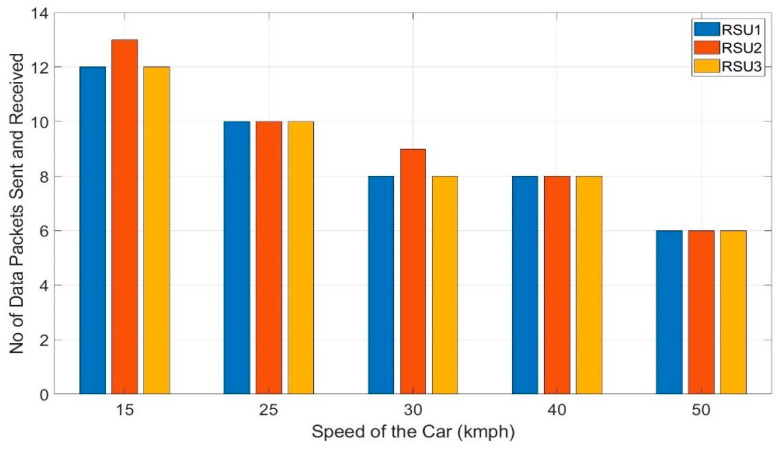
Data points received by each RSU at different speeds of the car *with* Kalman filter.

The same test is repeated with the Kalman filter in the algorithm as proposed and the number of data packets received by each RSU at a different speed is presented in Figure 14. It shows that the number of data packets received by RSU1, RSU2, and RSU3 at any particular speed is almost the same which depicts the smooth handoff of the RSUs by the data sending vehicle. This is because the Kalman filter stabilized the RSSI values removing any unintended fluctuations of the values. For the speeds, 25, 40, and 50 kmph, each of RSU 1, 2, and 3 received an equal number of data packets –10 data packets at 25 kmph, eight data packets at 40 kmph, and six data packets at 50 kmph. Still, there are some discrepancies, at 15 kmph, both RSU 1 and RSU 3 received 12 data packets each whereas RSU 2 received 13 and at 30 kmph, RSU 1 and 2 received eight data points each, but RSU 2 received eight. With the Kalman filter, the variation of the number of data packets improved by 5.16% and 15.36% for 15 and 30 kmph, respectively. At 25, 40, and 50 kmph, the transition among the RSUs is perfect with 0% variation for each of them where they improved the variation of the number of data packets by 52.84%, 7.87%, and 30.28%, respectively. 

The small percentage of discrepancies that are still present might be caused by the asymmetrical movement of the traffic and the erratic behavior of the radio signal. At 15 kmph, RSUs received a total of 37 data packets which imply the reception of one data packet every 25.9 m. Similarly, with 25 kmph and 30 kmph, each data packet is received in every 32 m and 38.4 m. This clearly shows a regular pattern of increased distance to receive one packet of data from the vehicle. So, with a higher speed like 40 kmph and 50 kmph, the OBU can transmit a data packet every 40 m and 53.34 m respectively with smooth transitioning between the receiving RSUs which denotes the stability and reliability of the proposed architecture. It is mention-worthy that all the data packets sent by the OBU are successfully received by RSU 1, 2, and 3 with no loss of data packet for both of the tests- without Kalman filter and with Kalman filter. Therefore, the achieved packet delivery ratio of the proposed architecture under a practical scenario is 100%. It denotes the reliability and the robustness of the architecture. The OBU takes an average of 4.57 s for completing one cycle, thus to collect, parse, and send the data to the RSU and other vehicle defined by the algorithm 1 and 2. This time period is very important for the real-time applications of the V2X. This time period can be improved further by implementing the architecture with the processing unit of higher clock speed and higher memory. 

### 6.3. Statistical Analysis of Proposed System

The aspect of the number of the data points received RSUs along the road needs to be verified since two manipulating factors—order of the RSU and different speeds of the car—are considered in the performance evaluation. As the groups have different spreads, i.e., number of the data points are different for different RSU and speeds, parametric tests are used for statistical analysis [75]. Therefore, the statistical analysis using analysis of variance (ANOVA) is performed with test data to determine how the order of the RSU and speed of the vehicles affect the total number of data packets received by any RSU. Thus, the following assumptions were made for statistical analysis purposes: First, it is assumed that the variation in RSU order will not produce diverse results with respect to the number of data packets received. Further, the tests were randomized to prevent unspecified disturbances that are associated with location, roadside infrastructures, or direct line of sight from being confounded with effects. This allowed for the assumption that errors are independently distributed random variables. 

A hypothesis is made to determine the effect of the positioning order of the RSU (P-RSU) on the total number of data packets (ND) received by each of them. The speed of the vehicle (VS) remains constant in any single run for all of the RSUs. It is also assumed that all the RSUs receive the same ND irrespective of the P-RSU. With this assumption, a few hypotheses were formulated, including:

- Overall
  -- Null Hypothesis (H_0_): ND on different P-RSU will not vary.
  -- Alternative Hypothesis (H_A_): Some RSU will receive higher ND than others.
- Speed Effects
  -- Null Hypothesis (H0): The speed of the vehicles do not have any effect on the total number of data packets. All of the treatment effects, in this case, are equal to zero. i.e., τ1 = τ2 = τ3 = 0.
  -- Alternative Hypothesis (HA): At least of the speeds has a significant effect on the total number of data packets. i.e., at least one of τ ≠ 0.
- Interaction Effects
  -- Null Hypothesis (H0): None of the interactions have any effect on the flow rate.
  -- Alternative Hypothesis (HA): At least one interaction has a significant effect on the flow rate.

The data analysis started with the assumption that the interaction of P-RSU and VS does not have a significant effect on the ND. A study was performed to identify the various methods by which the significance of interactions can be found to find this interaction effect. Two factorial design with replication is determined to be the test method as the data are collected in multiple trials and as there were two factors in the test- P-RSU and VS. The data used to perform these two factorial tests is shown in Table 4 for three different P-RSU (RSU1, RSU 2, RSU 3) and five different VS (15 kmph, 25 kmph, 30 kmph, 40 kmph, 50 kmph). A general factorial design test is performed on the above data and Table 5 depicts the ANOVA results.

The results show that *p*-values for P-RSU and interaction are 0.611496 and 0.59726 which are greater than the assumed α = 0.05. These results suggest accepting the null hypothesis for both P-RSU and interaction effects. Therefore, the position of RSU and its interaction with vehicle speed do not have any significant effect on the number of data packets received by any of the RSUs. The *p*-value of the VS is less than the assumed α which suggests rejecting the null hypothesis. This implies that different vehicle speeds have effects on the number of data packets received by RSUs which is obvious. Thus, these results clearly imply the statistical significance of the performed tests and collected data. The aim of the statistical analysis in this work was to show that the position and order of the RSU do not adversely affect the number of data packets received by those RSUs but it might be affected by the speed of the vehicles going along the road. That said, if the vehicle does not change the speed and it goes alongside multiple RSUs for equal time or distances for each one of them, then every RSU will get an equal number of data packets.

### 6.4. Power and Energy Analysis

As the proposed architecture facilitates modularity, the existing vehicles do not need any extra circuitry or change in vehicle infrastructure to be enabled with V2X. These vehicles can keep the OBU on board and power it up with a small battery to be in the designed architecture. As it is designed to provide legacy vehicles with V2X communication capability, energy efficiency is another major concern. To improve energy efficiency, the units are designed with low power micro-controller chips and LoRa transceivers. Table 6 and Table 7 denote the power consumption and energy consumption respectively for both unencrypted and encrypted data transmission. The table shows that the average power consumption of OBU with unencrypted and encrypted data is 341.2 mW and 346.15 mW respectively. This power consumption is very less compared to the task it is performing. On the other hand, RSU consumes less than half of the power that OBU consumes.

The power consumption of the RSU is 164.3 mW and 169.25 mW, respectively, with unencrypted and encrypted data communication. The energy consumption for each packet of data transmission is also very efficient. The encrypted communication of each data packet draws the energy of 0.037 J and 0.018 J respectively for OBU and RSU. With a battery of 3.7 V and a capacity of 10,000 mAh with 10% discharge safety, the driving hours with a single charge can be calculated by (3). The running hours of both OBU and RSU are presented in Table 8.

(3) $$Driving\;hours=\frac{\mathrm{BatteryCapacity}-\left(\mathrm{BatteryCapacity}\times \mathrm{DischargeSafetyRate}\right)}{\mathrm{Average}\;\mathrm{Current}\;\mathrm{Consumption}}$$

These results reflect excellent energy efficiency for both OBU and RSU. The calculated running time of the OBU are almost half of the RSU as the OBU is equipped with sensors causing it to draw more current. Even though with encrypted data transmission, an OBU can offer up to 130 h of driving time with a 1000 mAh, 3.7 V battery.

On the contrary for the same setup, an RSU can operate up to 266 h with a single charge of the battery, so if a person drives for two hours on average, a 10,000 mAh battery can support an OBU for more than two months before the battery needs to be replaced whereas RSU can last more than 4 months. The proposed system is also compared with the other state-of-the-art LoRa V2X models in Table 9 to analyze important functionalities of the architecture like- reliability, latency, energy, security, prototype designing, and evaluation of the systems. The table shows that none of the literature addressed all these functionalities presented. For example, some of the works focused on architectural reliability and latency, whereas they do not account for vital aspects such as evaluation under practical scenarios or statistical analysis which would provide a significant, insightful, and complete analysis. In comparison to these LoRa V2X models, the proposed architecture provides more detailed works which are also evaluated under different practical scenarios and with statistical analysis. Advantages of the proposed LoRa V2X in comparison with the state of the literature include:

- more functionalities than any other LoRa V2X architecture.
- achieved higher reliability with lower latency under practical scenarios.
- energy-efficient and compact, and has the potential to integrate with legacy vehicles.
- extensively tested under practical scenarios with vehicles on the move at different speeds and the test data shows statistical significance.

## 7. Conclusions

Communication technologies like LoRa play an important role in developing a reliable and secure communication architecture for V2X. It can offer both reliability and modularity for the prevailing vehicles with no additional circuitry. Moreover, it also addresses challenges like security and power consumption. This paper presents a reliable and robust architecture for V2X communication with LoRa which involves the design, implementation, and real-world testing with the prototypes. It addresses some of the fundamental challenges such as reliability, data handover, time criticality, modularity, and energy efficiency with vehicles on the move under different practical scenarios which is lacking in the existing LoRa V2X literature. This research work also helps to fill up the void in the literature regarding robust architecture and evaluation of V2X under real traffic scenarios. The results show the suitability of the LoRa architecture for V2X communications where mobility of the vehicles presents some unique challenges. This work inspires other researchers to work further and improve LoRa-based V2X as it has the potentiality to bring revolution in ITS applications.

## Figures and Tables

**Table 1:** Comparison of different wireless technologies used in V2X.

Wireless Technology	Network Type	Spectrum	Transmission Range	Transmission Throughput	Mobility Support
LoRa [48]	WAN	433, 868 & 915 MHz	2–5 km (Urban) and 15 km (suburban)	27 Kbps	Yes
Zigbee [49]	PAN	433, 868 & 915 MHz	10–100 m	250 Kbps for 2.4 GHz; 40 Kbps for 915 MHz; 20 Kbps for 868 MHz	Yes
DSRC [42]	Wireless Ad-hoc	5.8‒5.9 GHz	1 km	2.5 Mbps	Yes
Bluetooth Low Energy (BLE) [50]	PAN	2.4 GHz	100 m (Class 1 device); 10 m (Class 2 device); 1 m (Class 3 device)	1400 Kbps (BLE 5)	Limited
WiMAX [51]	Wireless Broadband	5.8 GHz	50 km	70 Mbps	Yes
C-V2X [52]	Cellular Wireless Broadband	5.9 GHz	>DSRC & LoRa	>DSRC & LoRa	Yes

**Table 2:** Research focus, wireless technologies, and limitations of different LoRa-based vehicular networks.

Research	Wireless Technology	Research Focus	Limitation
Performance evaluation of LoRa in V2X [58]	LPWAN technology- LoRa	Design and implementation of LoRa based V2X	Tested only with low traffic density scenario
LoRa and eMTC-based V2X [59]	LoRaWAN and eMTC	LoRaWAN and eMTC based V2X with Monte Carlo simulation	Not tested under real road scenarios
Vehicular ecosystem with LPWAN [60]	LoRaWAN	LPWAN-based V2V and V2X communication	Emphasized on notifying traffic incidents only
Secure V2V/V2I communications [61]	LoRa	RSSI-based secure key generation scheme for V2X	Tested only in the indoor testbed.
Autonomous V2X network [12]	LoRaWAN	Reducing network latency and data size for the V2X	No direct communication among vehicles or vehicle to infrastructure
LoRan-based vehicle charging architecture [62]	LoRa	Communication protocol between electric vehicles and charging stations	Designed only for Vehicle to Grid (V2G)
Software-defined vehicular communication with LoRa [63]	LoRa	Closed subnet service based on software-defined internet of vehicles	Evaluated only with simulations.
Vehicle monitoring with LoRaWAN [64]	LoRaWAN	End-to-end architecture for vehicle monitoring	Higher speeds of the vehicles are not addressed.
V2V & V2I with LoRaWAN and BLE [65]	LoRaWAN & BLE	Framework of V2V and V2I with LoRaWAN and BLE	Mobility issues under practical scenarios are not considered.
Vehicle tracking system with LoRa [66]	LoRaWAN	Public vehicle tracking system.	Not adequate for real-time V2X communication.
Drone-based vehicle monitoring [38]	LoRaWAN	Drone-based LoRaWAN access point	Poses higher chances of network failure due to drone-based access points.

**Table 3:** Different SF and the corresponding parameters [74].

SF	Data Rate (kbps)	Time-on-Air (ms)	Receiver Sensitivity (dBm)	SNR (dB)
7	5.47	36	−123	−6
8	3.13	64	−126	−9
9	1.76	113	−129	−12
10	0.98	204	−132	−15
11	0.54	365	−134.5	−17.5
12	0.29	682	−137	−20

**Table 4:** Data used in two factorial design tests.

P-RSU	VS				
	15 kmph	25 kmph	30 kmph	40 kmph	50 kmph
	ND				
RSU 1	12	10	8	8	6
	13	10	9	8	6
	12	10	8	8	6
RSU 2	13	11	8	8	6
	13	10	9	8	5
	12	10	9	8	6
RSU 3	12	10	8	8	6
	12	10	9	8	6
	12	10	8	8	6

**Table 5:** ANOVA table for two factorial design.

Source of Variation	SS	df	MS	F	*p*-Value	F crit
SS P-RSU	0.177778	2	0.088889	0.5	0.611496	3.31583
SS VS	212.3111	4	53.07778	298.5625	1.08E-23	2.689628
SS Interaction	1.155556	8	0.144444	0.8125	0.59726	2.266163
SS Within	5.333333	30	0.177778			
Total	218.9778	44				

**Table 6:** Power consumption for data transmission.

Unit	Unencrypted Data (mW)			Encrypted Data (mW)		
	Max.	Min.	Mean	Max.	Min.	Mean
OBU	681.3	303.3	341.2	703.3	322.7	346.15
RSU	505.45	123.1	164.3	598.9	123.1	169.25

**Table 7:** Energy consumption for data transmission.

Unit	Unencrypted Data (J)	Encrypted Data (J)
OBU	0.034	0.037
RSU	0.017	0.018

**Table 8:** Running time of OBU and RSU with 3.7 V, 1000 mAh battery.

Unit	Unencrypted Data (hours)	Encrypted Data (hours)
OBU	131.89	130.00
RSU	273.89	265.88

**Table 9:** Comparison of the proposed architecture with state-of-the art LoRa V2X models.

Name of the Model	Functionalities							
	Reliability	Latency Analysis	Energy Analysis	Security	Prototype Designing	Evaluation Under Practical Scenarios	Evaluation with Different Speeds	Statistical Analysis
Evaluation of LoRa in V2X [58]	✓	✓	✗	✓	✓	✓	✗	✗
LoRa and eMTC- based [59]	✓	✗	✗	✓	✗	✗	✓	✗
Vehicular eco- system [60]	✗	✗	✗	✗	✓	✓	✗	✗
Secure V2V/V2I [61]	✗	✗	✗	✓	✓	✓	✗	✗
V2X network [12]	✓	✓	✗	✓	✓	✓	✗	✗
Vehicle charging architecture [62]	✓	✗	✗	✓	✓	✗	✗	✗
Software defined LoRa V2X [63]	✓	✗	✗	✗	✓	✗	✗	✗
V2V and V2I with LoRaWAN and BLE [65]	✓	✗	✗	✗	✓	✗	✗	✗
Drone-based LoRa V2X [38]	✓	✓	✓	✗	✗	✗	✗	✗
Proposed	✓	✓	✓	✓	✓	✓	✓	✓
